# Supplementary material for: A novel framework for quantifying the clinical impact of substandard and falsified antimicrobials: Application to typhoid fever
Source: PLOS Glob Public Health. 2026 Aug 31;6(8):e0007207. doi: 10.1371/journal.pgph.0007207 (PMC13529222; doi:10.1371/journal.pgph.0007207)
Supplement: Supplementary file 1 [file pgph.0007207.s001.pdf]

S1 Appendix

Text A: additional methods and results for stool positivity outcome

Additional methods. Stool samples that were positive following the completion of treatment could give an indication of the potential for onward transmission. For this outcome, 88/572 (15%) patients did not have a stool sample taken and so were removed from the analysis. For a further 67 (12%) patients the number of positive stool samples taken was not recorded, and instead it was recorded whether at least one sample was positive. For all included patients the total number of stool samples tested was recorded. With this in mind, we used a mixed likelihood function, that is,

$$\mathscr{L}(p_i|n_i, r_i) = \binom{n_i}{r_i} p_i^{r_i} (1 - p_i)^{n_i - r_i}$$

if r_i is known, and

$$\mathscr{L}(p_i|n_i, r_i > 0) = 1 - (1 - p_i)^{n_i}$$

if r_i is unknown, where r_i represents the number of positive stool samples, n_i the total number of stool samples, and p_i the probability of a positive stool sample predicted by the model and its parameters.

Results. To investigate the effect of antibiotic dose on potential for onward transmission, we also examined an additional outcome: stool positivity following the completion of treatment. Excluded patients had a higher average total dose than included patients (54.2 mg/kg (90% CI: 51.2 – 57.3) *vs.* 39.6 mg/kg (90% CI: 34.6 – 44.6) respectively) and a higher average MIC (0.178 mg/L (90% CI: 0.156 – 0.200) *vs.* 0.0879 mg/L (90% CI: 0.0620 – 0.114)). Due to the reduced data set and relatively low number of positive samples, it was difficult to conclude anything about the effect of dose on stool positivity (Fig S1), although the trend was in the direction of improved outcomes at higher doses when the infecting strain had a high MIC.

Text B: Additional details on alternative models

Methods. To examine the robustness of our results to our modelling choices, we also undertook the analysis with three alternative models, each of which are consistent with the causal assumptions described in Fig 2. These alternative models are listed in Table S2. Briefly, in Model S1 we divided daily dose by MIC prior to inclusion in the model, and included an interaction between this term and duration of treatment; in Model S2 we included a three-way interaction between daily dose, MIC, and duration of treatment; in Model S3 we included interactions between dose and duration of treatment and dose and weight, but excluded MIC from the model.

Results and discussion. For Model S1 we divided daily dose (mg/kg/day) by MIC (mg/L) before including it in the model and include an interaction between this term and duration of treatment. In this case, increasing dose/MIC led to clear reductions in both fever clearance time and treatment failure probability at all treatment durations (Figs S6 and S7). The effect was particularly strong when dose/MIC was high (i.e., when dose is low and MIC high), which is commensurate with our baseline finding that lower doses led to worse outcomes when MIC was high. Model S2, which includes a three-way interaction between daily dose, MIC, and treatment duration, showed a decrease in failure probability at high doses, but did not show a relationship between fever clearance time and dose at any MIC (Figs S8 and S9), counter to our findings with the baseline model and model S1. However, model S2 had a worse predictive performance than the baseline model on fever clearance time, as approximated by the leave-one-out information criterion (LOOIC; Table S3). Model S3, which does not include MIC, has no clear relationship between dose and the outcomes of interest (S10 and Fig S11s). In all three of these alternative models there is little evidence of an interaction between duration and dose. Comparing all models' predictive performance using LOOIC, the baseline model and model S1 had the best predictive performance on fever clearance time, the baseline model and model S2 had the best predictive performance on treatment failure, and model S3 was worst on both outcomes (Table S3)

Table A: Summary of included trial arms. Details are given only for ofloxacin arms. Where more than one arm of a study included ofloxacin treatment, the study has two rows; the arms can be distinguished by differing durations of treatment allocated.

**Journal article**	**Treatment Duration (days)**	**Ofloxacin Dose**	**Number of patients**	**Sex** (% female)	**Age** (years) median (IQR)	**Bodyweight** (kg) median (IQR)	**MIC** (mg/L) median (IQR)
Smith *et al.*, *Antimicrobial Agents and Chemotherapy* (1994)[16]	5	400 mg/day	19	31.6	21 (18–23)	46.0 (36.0–48.5)	1/32 (1/32–1/32)
Vinh *et al.*, *Antimicrobial Agents and Chemotherapy* (1996)[13]	3	15 mg/kg/day	52	48.1	9 (5–12)	18.4 (13.0–24.6)	1/16 (1/32–1/16)
	2	15 mg/kg/day	50	40.0	8 (6–11)	19.8 (14.5–23.9)	1/16 (1/32–1/16)
Chinh *et al*. *Transactions of the RSTMH* (1997)[17]	3	10 mg/kg/day	55	52.7	24 (18.5–27.5)	45.0 (41.0–48.0)	1/16 (1/32–1/16)
	2	15 mg/kg/day	46	65.2	23 (19–30)	46.0 (43.0–48.0)	1/16 (1/32–1/16)
Cao *et al*. *The Pediatric Infectious Disease Journal* (1999)[14]	5	10 mg/kg/day	68	44.1	6.5 (5–10)	17.0 (14.0–20.3)	1/16 (1/16–1/16)
Chinh *et al*. *Antimicrobial Agents and Chemotherapy* (2000)[18]	5	400 mg/day	45	44.4	24 (19–28)	47.0 (44.0–54.0)	1/8 (1/32–1/2)
Vinh *et al*. *Annals of Tropical*	3	10 mg/kg/day	89	44.9	8 (6–10)	17.0 (13.5–20.0)	1/16 (1/32–1/16)

Paediatrics (2005)[12]	2	10 mg/kg/day	77	42.9	8 (6–11)	17.0 (14.0–21.0)	1/16 (1/16–1/16)
Parry *et al*. *Antimicrobial Agents and Chemotherapy* (2007)[15]	7	20 mg/kg/day	69	49.2	8 (7–11)	20.0 (15.0–24.0)	1 (1/2–1)

Table B. Alternative models.

**Model**	**Equation**	**Description**
S1	outcome ~ t2(dose_over_MIC, duration) + s(wt) + s(study, bs = “re”)	Divide dose by MIC. Includes interaction between this value and treatment duration.
S2	outcome ~ t2(dose, duration, MIC) + s(wt) + s(study, bs = “re”)	Three-way interaction between dose, treatment duration, and MIC.
S3	outcome ~ t2(dose, duration, wt) + s(study, bs = “re”)	Three-way interaction between dose, treatment duration, and weight. **No consideration of MIC.**

Table C. Models compared by leave-out-out information criterion (LOOIC). By this metric, models with a lower LOOIC are expected to have a greater out-of-sample predictive performance; we display the difference in LOOIC from the best model, and the standard error of the difference. LOOIC is an approximation of leave-one-out cross-validation (LOOCV) using Pareto smoothed importance sampling (PSIS), and is calculated in the brms package.

**Model**	**Fever clearance time**	**Failure probability**
Baseline model	0.0	0.0
S1 model	6.1 (11.2)	14.2 (6.4)
S2 model	12.4 (3.7)	1.6 (4.3)
S3 model	74.8 (19.4)	22.0 (9.0)

Table D. Model fit diagnostics for main fever clearance time and failure probability models

**Model**	**Maximum *R*-hat**	**Minimum bulk ESS**	**Minimum tail ESS**	**Divergences**
Fever clearance time	1.00	2103	2037	0
Failure probability	1.00	2703	2766	0

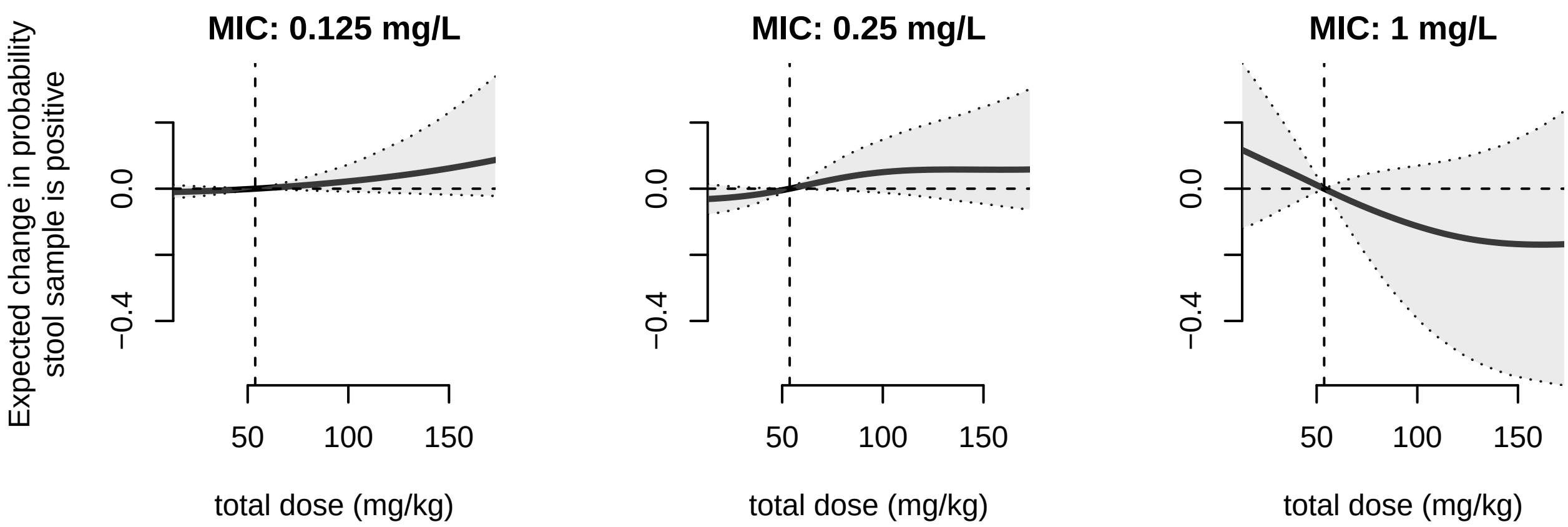

Fig A. Average treatment effect of different ofloxacin doses at different MICs (panels) on the probability of a positive stool sample compared to a baseline total dose of 52 mg/kg (vertical dashed line). Effects are only shown within the range of doses present in the data (13.8–172.4 mg/kg). All other parameters were kept at their baseline values for each individual. The grey shaded region represents a 90% credible interval. The horizontal dashed line represents no change from baseline.

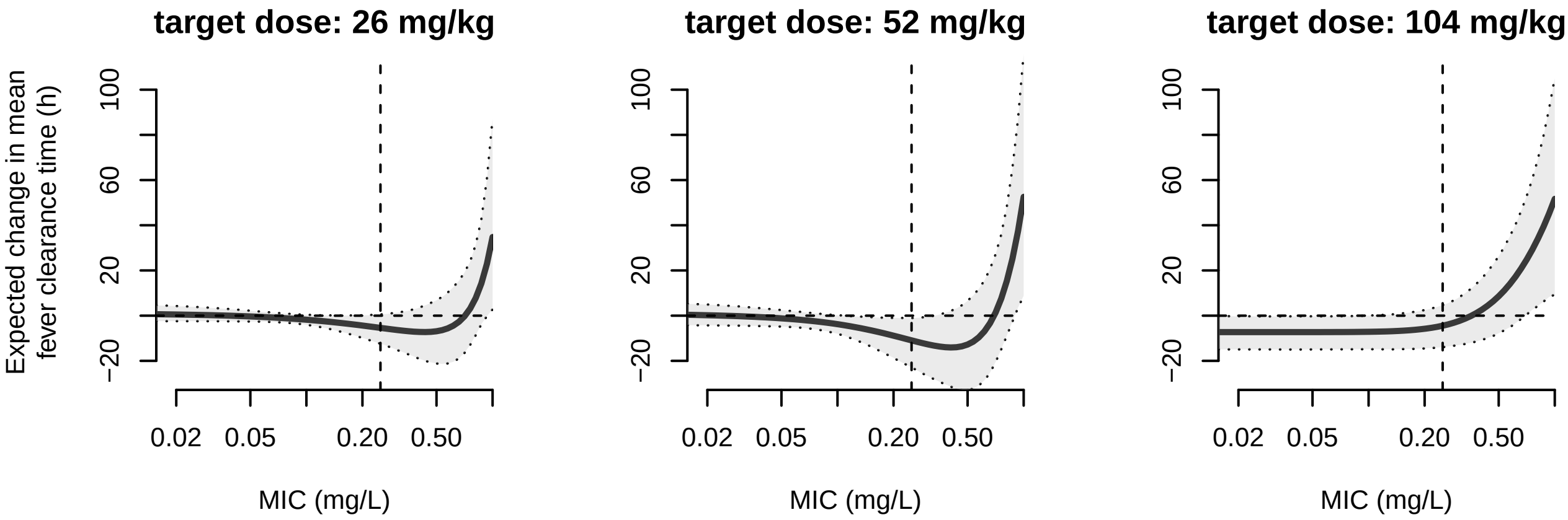

Fig B. Expected change in fever clearance time when patients take ofloxacin with %API distributed according to the ciprofloxacin distribution found in Bangladesh[27] *shown in Fig 3B compared to when they take ofloxacin with 100% API. Negative values indicate that the distribution decreased (i.e., improved) the fever clearance time. The shaded region shows 90% credible intervals. The panels show different target doses (i.e., the dose represented by 100% API). The vertical dashed line indicates the breakpoint for ofloxacin; MICs to the right of this line are considered non-susceptible.*

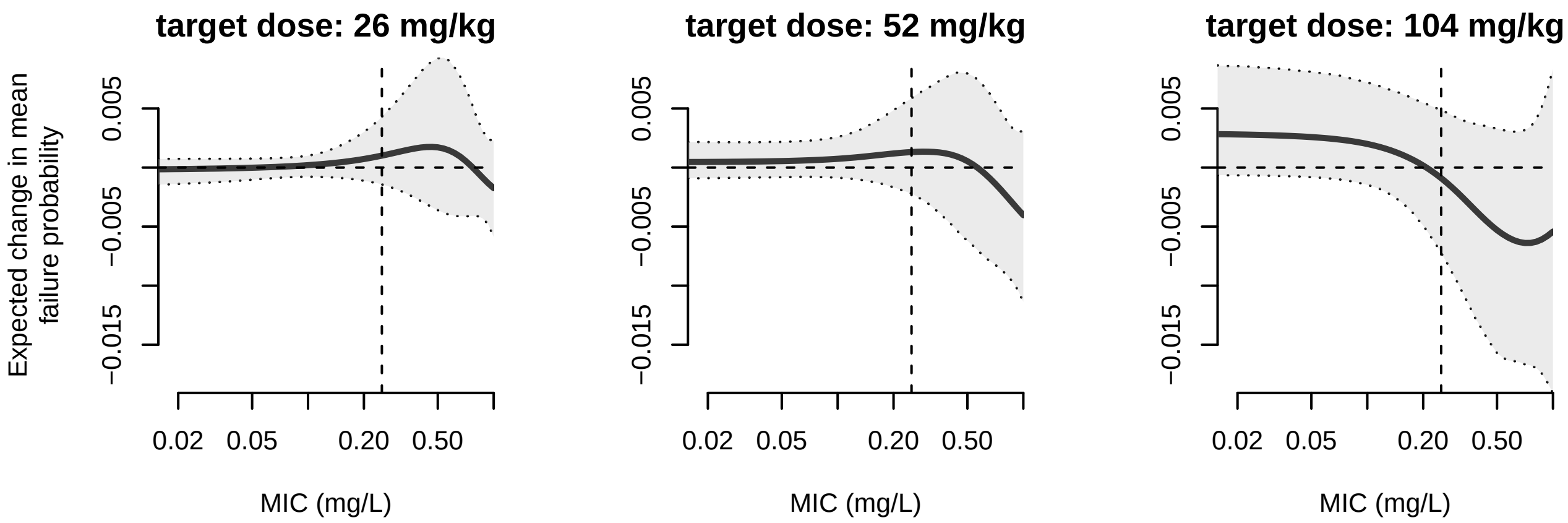

Fig C. Expected change in failure probability when patients take ofloxacin with %API distributed according to the ofloxacin distribution shown in Fig 3A compared to when they take ofloxacin with 100% API. Negative values indicate that the distribution decreased (i.e., improved) the fever clearance time. The shaded region shows 90% credible intervals. The panels show different target doses (i.e., the dose represented by 100% API). The vertical dashed line indicates the breakpoint for ofloxacin; MICs to the right of this line are considered non-susceptible.

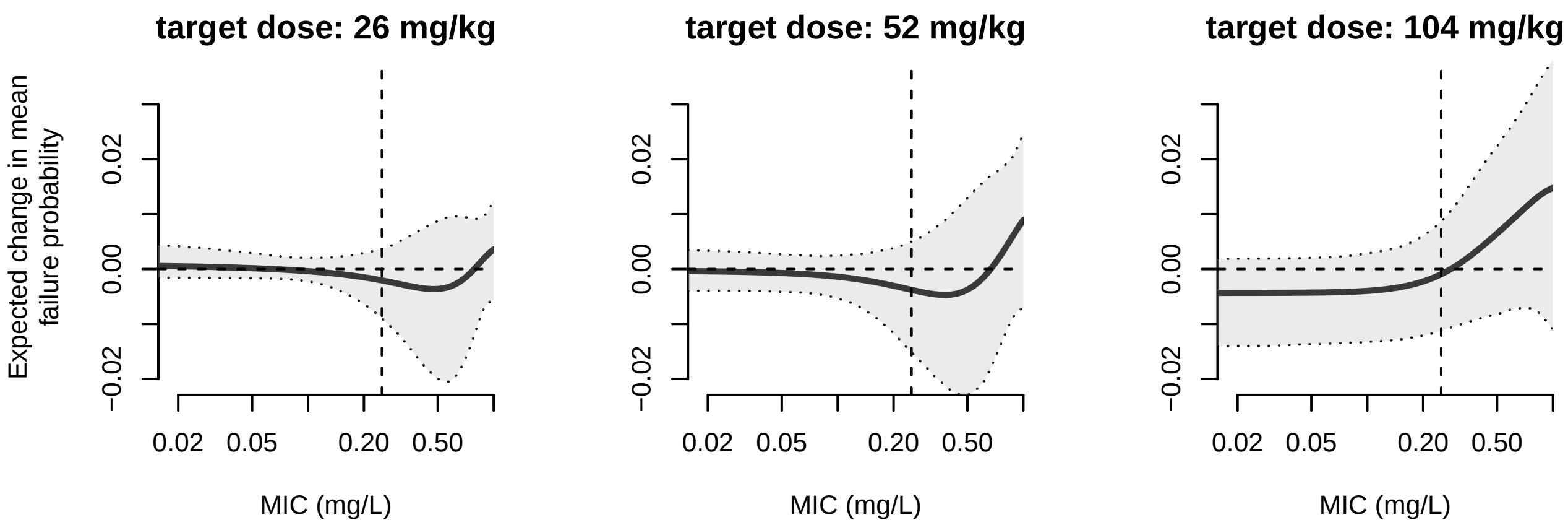

Fig D. Expected change in failure probability when patients take ofloxacin with %API distributed according to the ciprofloxacin distribution shown in Fig 3B compared to when they take ofloxacin with 100% API. Negative values indicate that the distribution decreased (i.e., improved) the fever clearance time. The shaded region shows 90% credible intervals. The panels show different target

doses (i.e., the dose represented by 100% API). The vertical dashed line indicates the breakpoint for ofloxacin; MICs to the right of this line are considered non-susceptible.

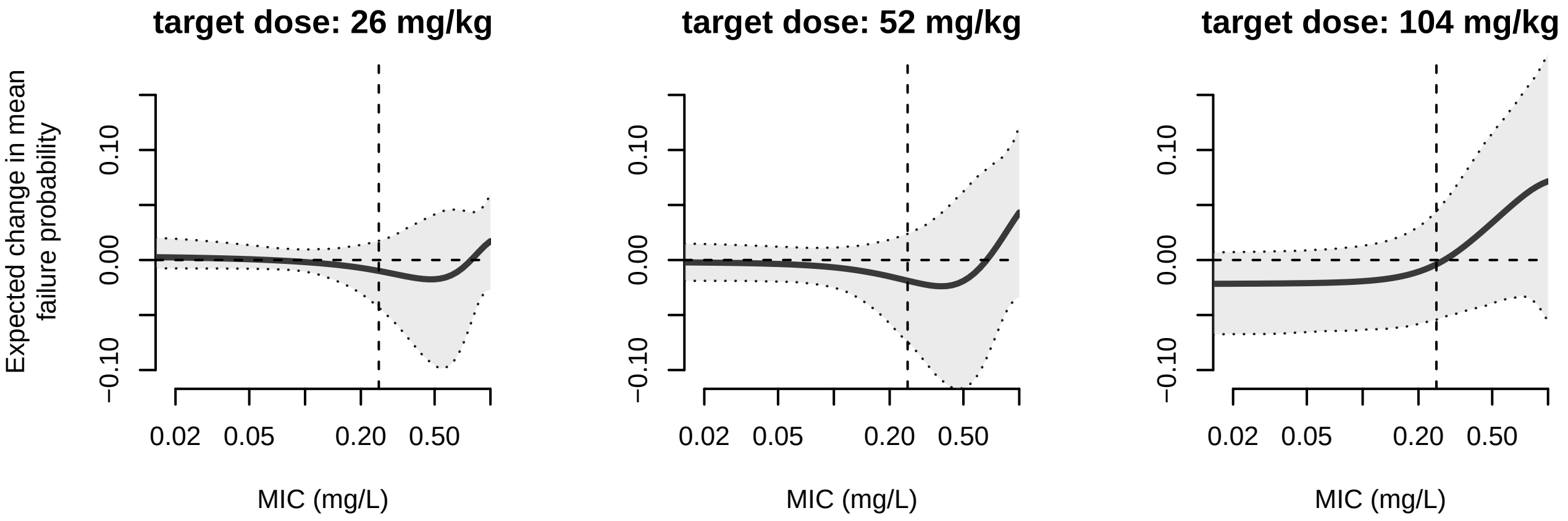

Fig E. Expected change in failure probability when patients take ofloxacin with %API distributed according to the ciprofloxacin distribution found in Bangladesh[27] shown in Fig 3B compared to when they take ofloxacin with 100% API. Negative values indicate that the distribution decreased (i.e., improved) the failure probability. The shaded region shows 90% credible intervals. The panels show different target doses (i.e., the dose represented by 100% API). The vertical dashed line indicates the breakpoint for ofloxacin; MICs to the right of this line are considered non-susceptible.

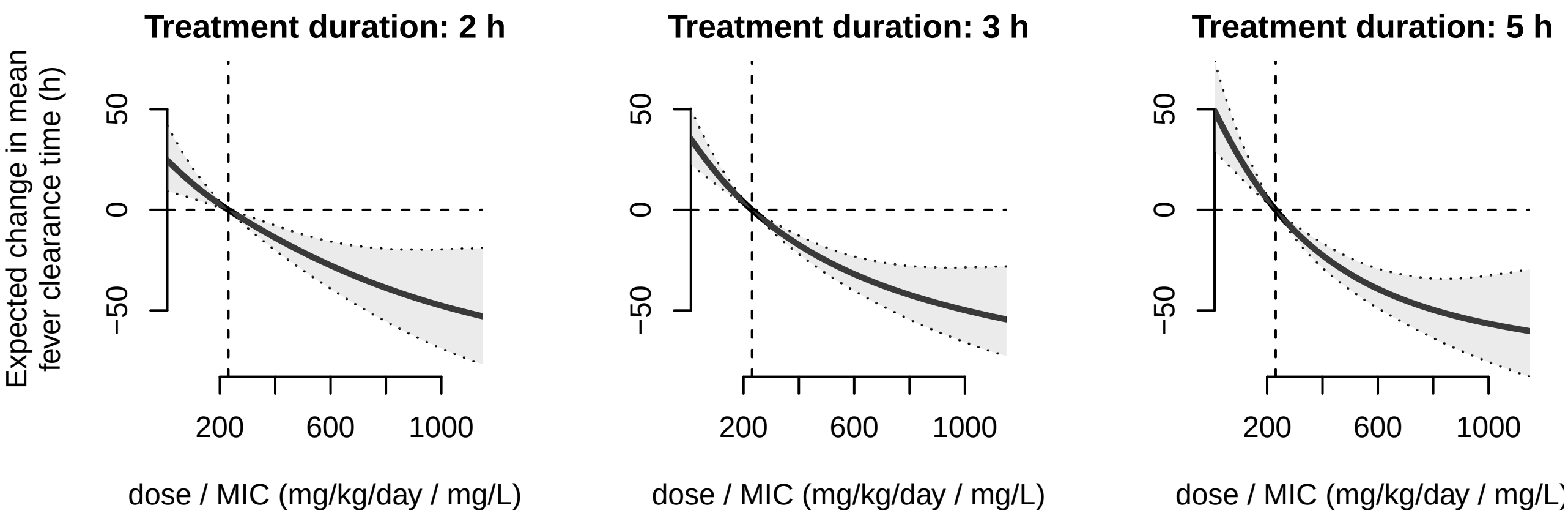

Fig F. Average treatment effect of different ofloxacin dose / MICs at different treatment durations (i.e., patients received the dose shown on the x-axis for the duration shown above the panels) on fever clearance time compared to a baseline dose / MIC of 230 mg/kg/day / mg/L (vertical dashed line) using model S1. Effects are only shown within the range of doses present in the data (13.8–172.4 mg/kg). All other parameters were kept at their baseline values for each individual. The grey shaded region represents a 90% credible interval. The horizontal dashed line represents no change from baseline.

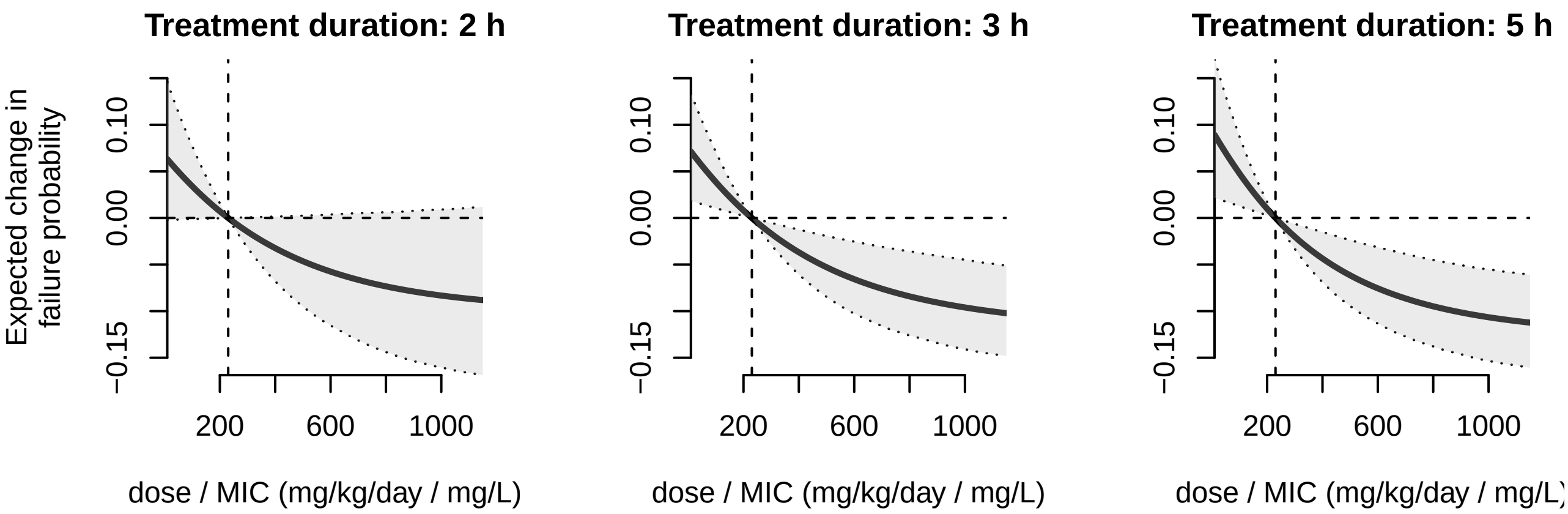

Fig G. Average treatment effect of different ofloxacin dose / MICs at different treatment durations (panels) on failure probability compared to a baseline dose / MIC of 230 mg/kg/day / mg/L (vertical dashed line) using model S1. Effects are only shown within the range of doses present in the data (13.8–172.4 mg/kg). All other parameters were kept at their baseline values for each individual. The grey shaded region represents a 90% credible interval. The horizontal dashed line represents no change from baseline.

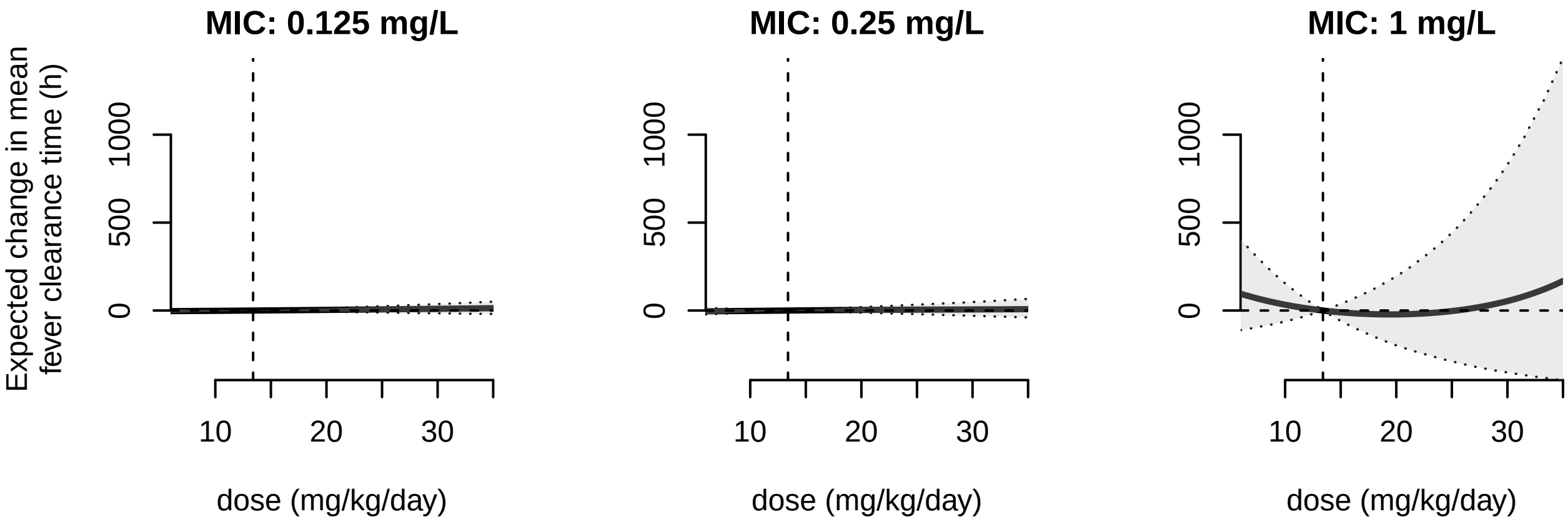

Fig H. Average treatment effect of different ofloxacin doses at different MICs (panels) on fever clearance time compared to a baseline dose of 14 mg/kg/day (vertical dashed line) using model S2. Effects are only shown within the range of doses present in the data (13.8–172.4 mg/kg). All other parameters were kept at their baseline values for each individual. The grey shaded region represents a 90% credible interval. The horizontal dashed line represents no change from baseline.

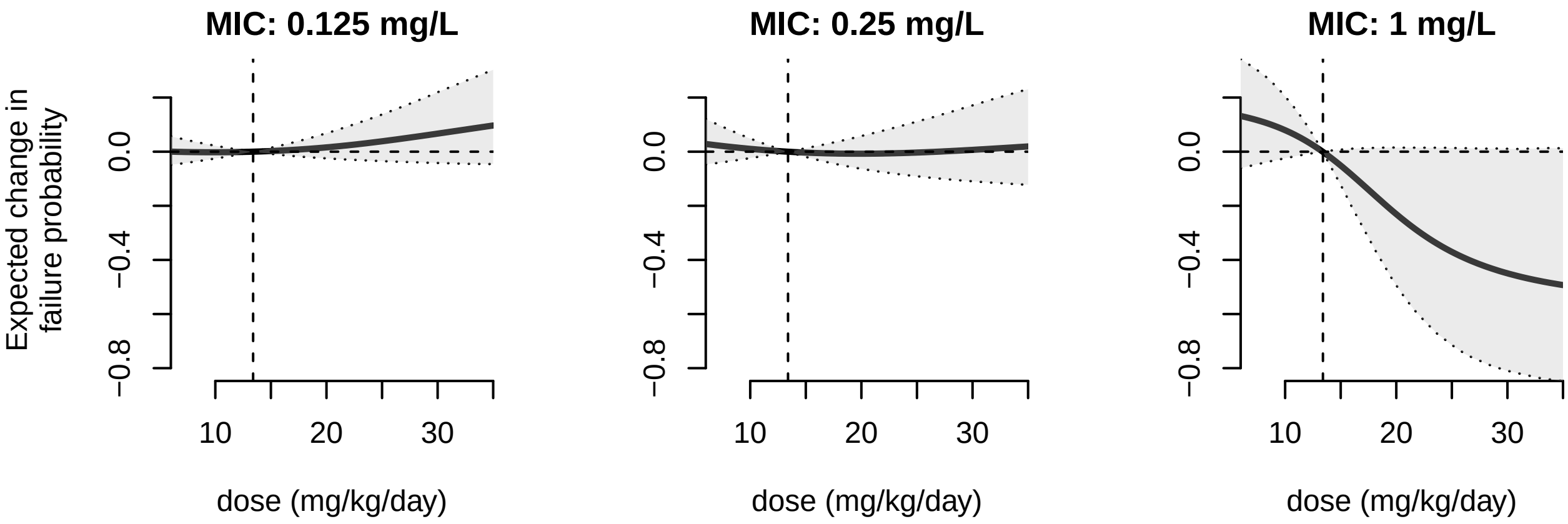

Fig I. Average treatment effect of different ofloxacin doses at different MICs (panels) on failure probability compared to a baseline dose of 14 mg/kg/day (vertical dashed line) using model S2. Effects are only shown within the range of doses present in the data (13.8–172.4 mg/kg). All other

parameters were kept at their baseline values for each individual. The grey shaded region represents a 90% credible interval. The horizontal dashed line represents no change from baseline.

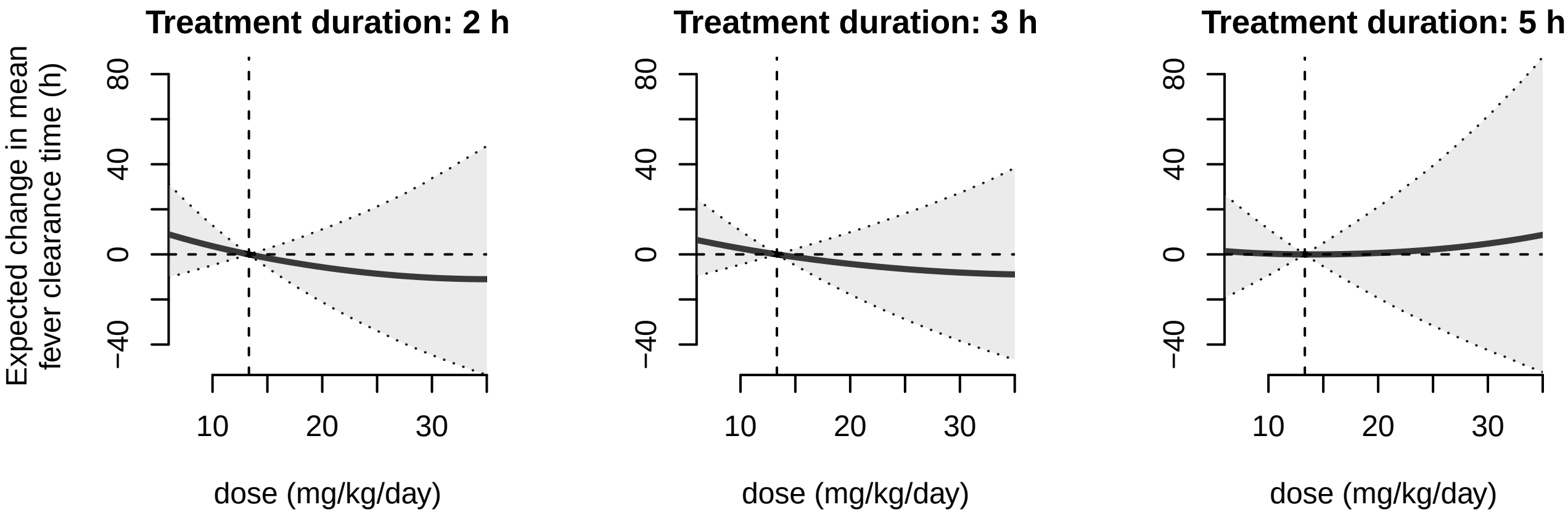

Fig J. Average treatment effect of different ofloxacin doses at fever clearance times (panels) on failure probability compared to a baseline dose of 14 mg/kg/day (vertical dashed line) using model S3. Effects are only shown within the range of doses present in the data (13.8–172.4 mg/kg). All other parameters were kept at their baseline values for each individual. The grey shaded region represents a 90% credible interval. The horizontal dashed line represents no change from baseline.

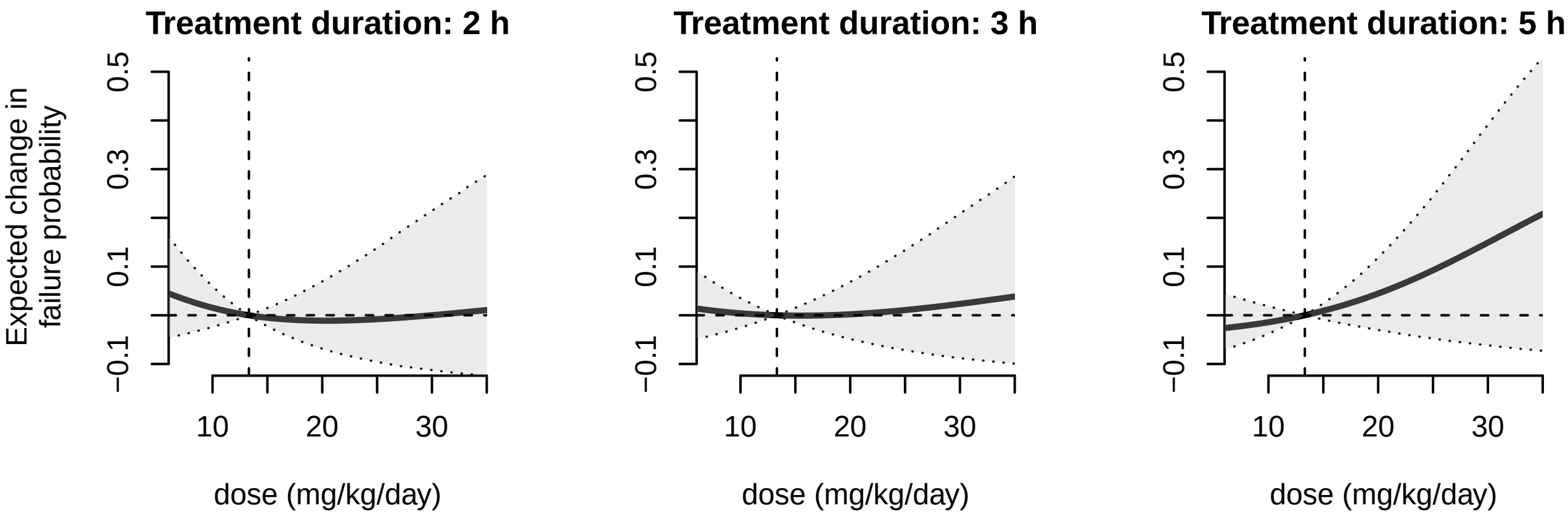

Fig K. Average treatment effect of different ofloxacin doses at treatment durations (panels) on failure probability compared to a baseline dose of 14 mg/kg/day (vertical dashed line) using model S3. Effects are only shown within the range of doses present in the data (13.8–172.4 mg/kg). All other parameters were kept at their baseline values for each individual. The grey shaded region represents a 90% credible interval. The horizontal dashed line represents no change from baseline.

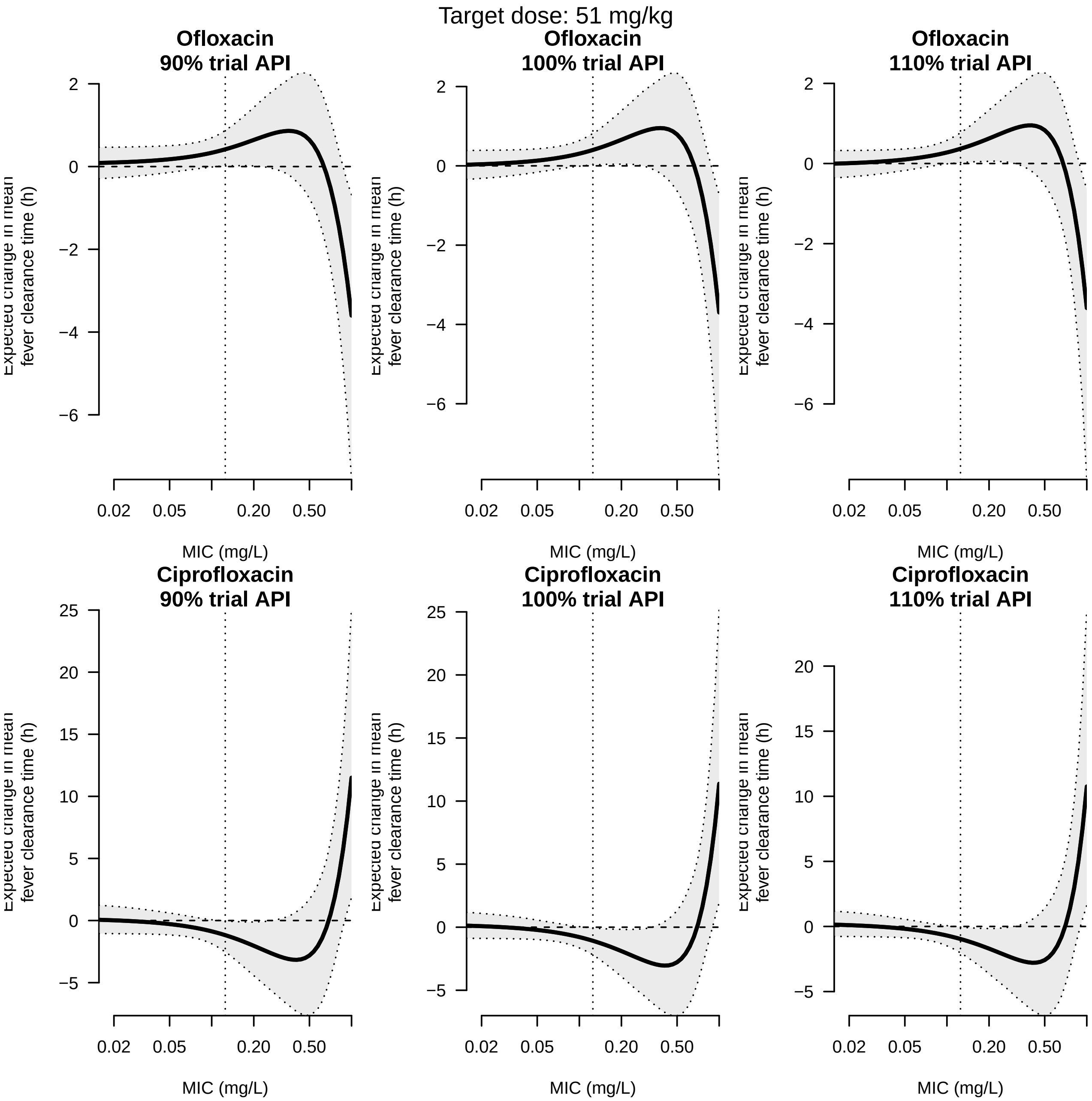

Fig L. Sensitivity to assumption that all trial medicines contained 100% of their stated API. Expected change in fever clearance time when patients take ofloxacin with %API distributed

according to the ofloxacin and ciprofloxacin distributions shown in Fig 3 compared to when they take ofloxacin with 100% API and assuming the medicines in clinical trials contained either 90%, 100%, or 110% of their stated API. The shaded region shows 90% credible intervals. The vertical dashed line indicates the breakpoint for ofloxacin; MICs to the right of this line are considered non-susceptible.

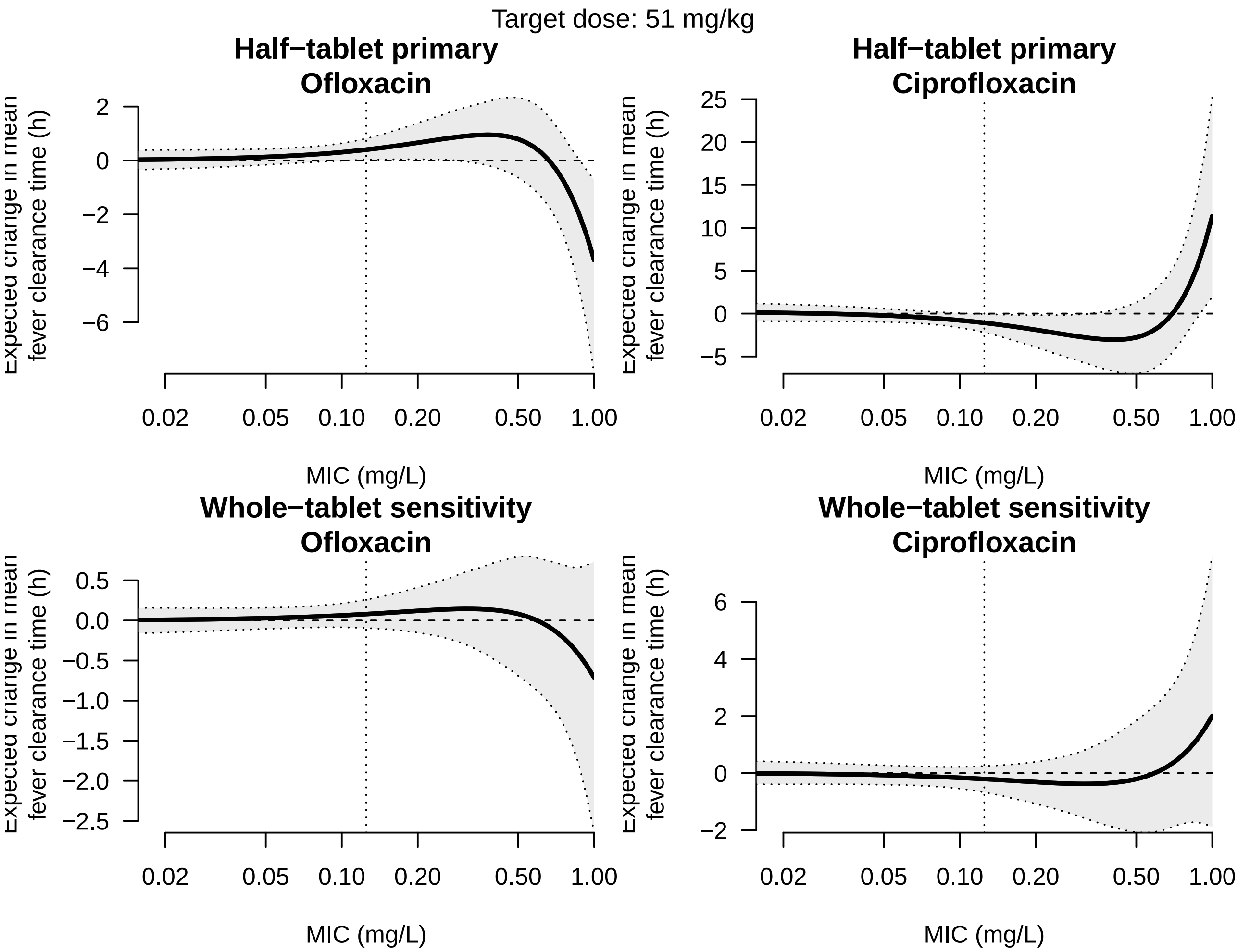

Fig M. Sensitivity to tablet rounding assumption. Plots show expected change in fever clearance time when patients take ofloxacin with %API distributed according to the ofloxacin and ciprofloxacin distributions shown in Fig 3 compared to when they take ofloxacin with 100% API and assuming the medicines in clinical trials were administered in whole-tablet increments. The shaded region shows 90% credible intervals. The vertical dashed line indicates the breakpoint for ofloxacin; MICs to the right of this line are considered non-susceptible.

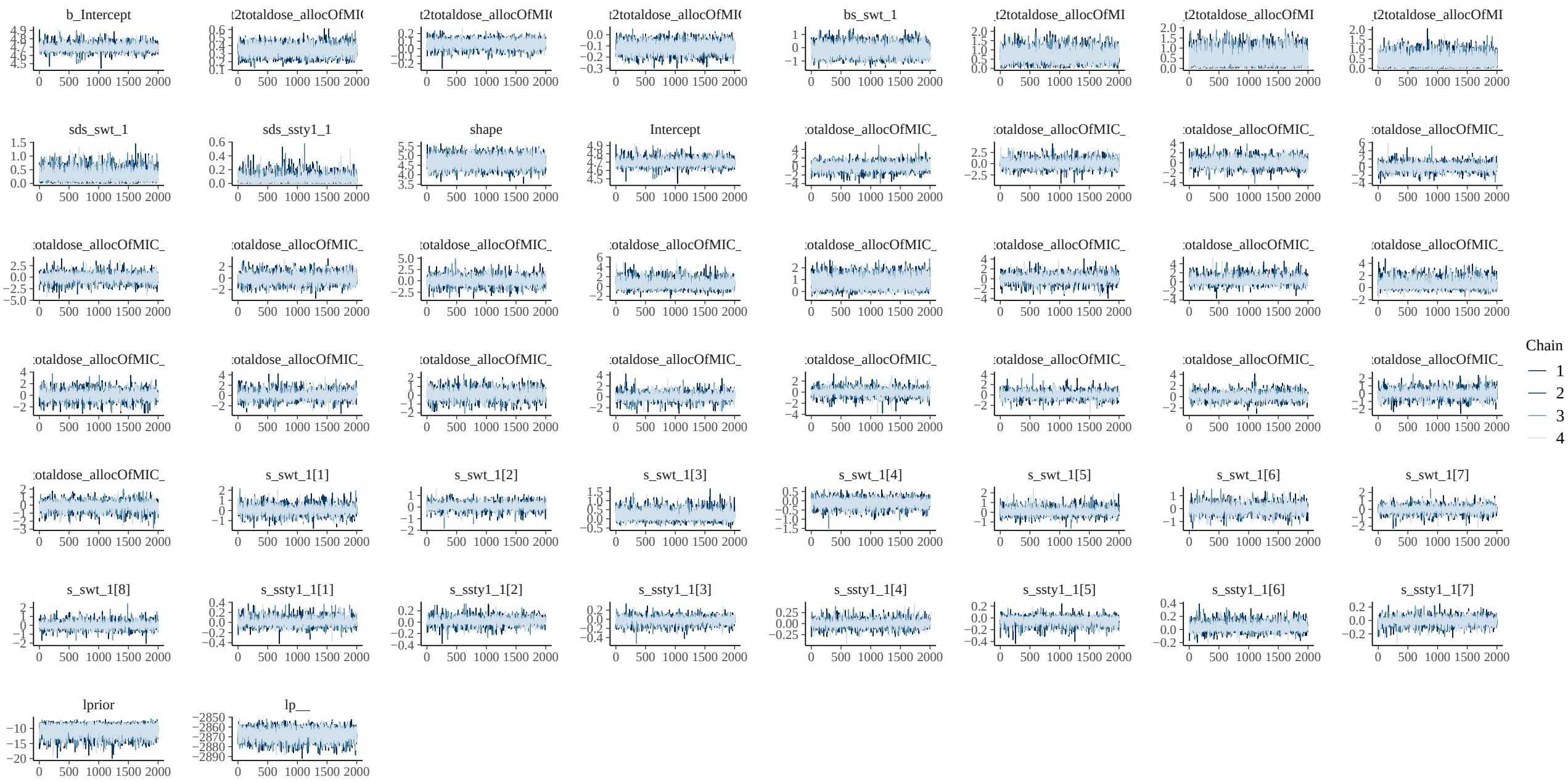

Fig N. Traceplots for main fever clearance time model.

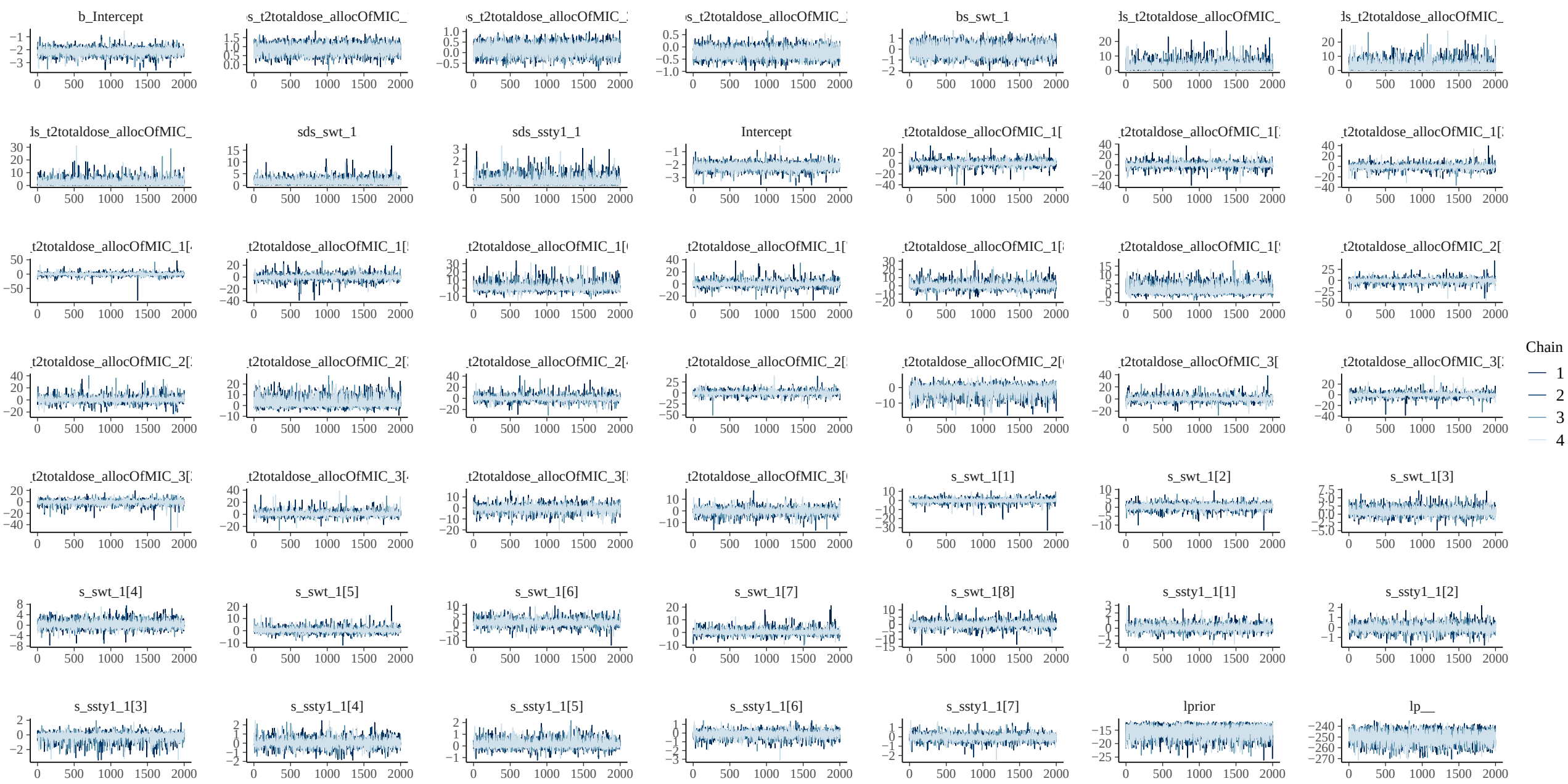

Fig O. Traceplots for main failure probability model.

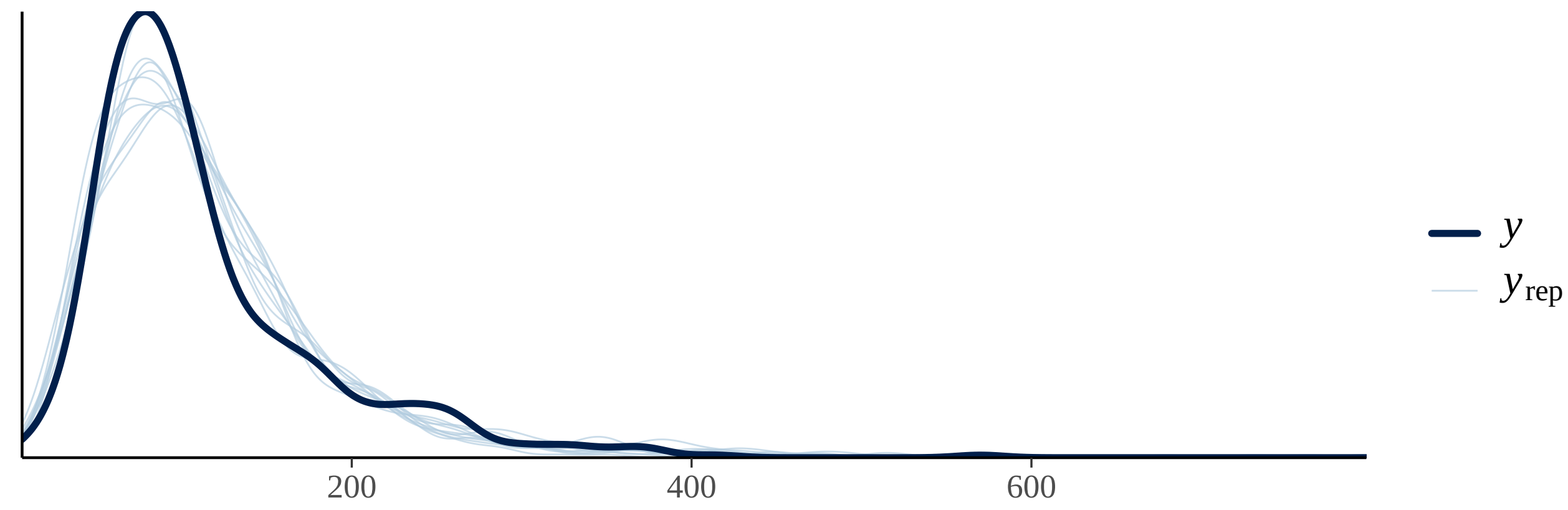

Fig P. Posterior predictive check for main fever clearance time model. Solid line shows distribution of fever clearance times (h, x-axis) in the data, blue lines show posterior draws of fever clearance time distribution.

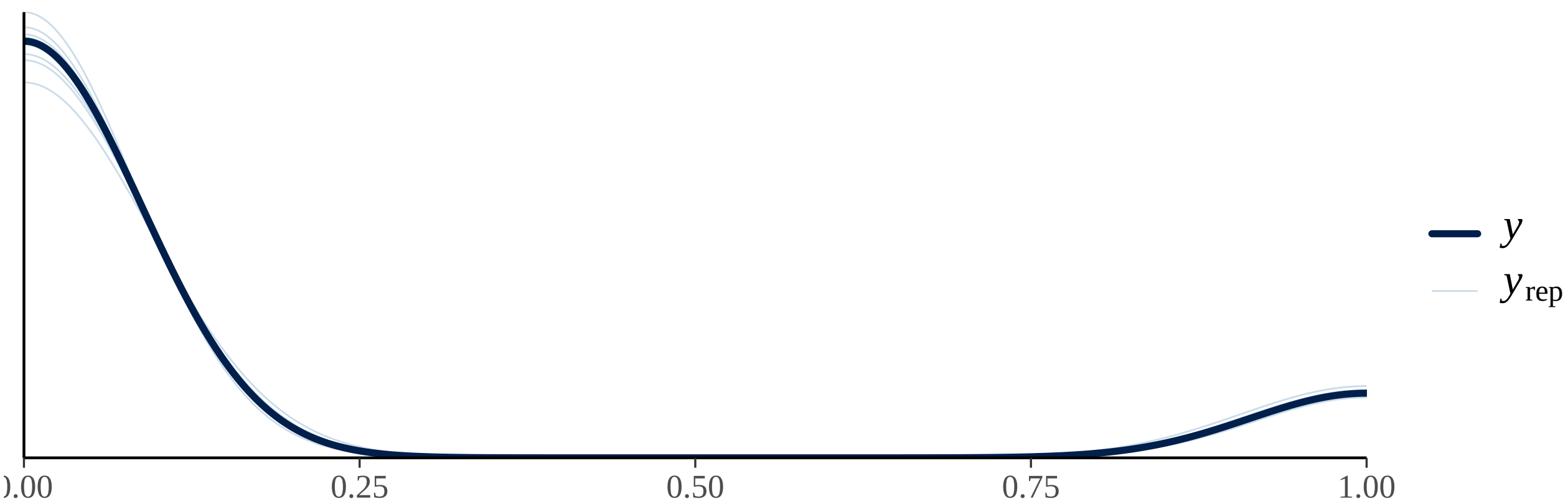

Fig Q. Posterior predictive check for main failure probability model. Solid line shows distribution of failures (x-axis) in the data, blue lines show posterior draws of failures distribution.
