# Supplementary material for: A novel framework for quantifying the clinical impact of substandard and falsified antimicrobials: Application to typhoid fever
Source: PLOS Glob Public Health. 2026 Aug 31;6(8):e0007207. doi: 10.1371/journal.pgph.0007207 (PMC13529222; doi:10.1371/journal.pgph.0007207)
Supplement: Supplementary file 2 [file pgph.0007207.s003.docx]

Response to reviewers

In the below, ordinary text denotes reviewer comments, **bold text** our responses, and quoted text passages added to the main document. Line numbers refer to the corrected version, not the tracked changes version.

Reviewer #1

This study is very interesting, and there are still few publications that link medicines quality with treatment outcomes. However, there are some comments to improve the information and discussion in the paper.

**Thank you for your kind words and helpful feedback. We have addressed each comment in turn below.**

R1C1

Line 33: of antimicrobials in low- and middle-income countries SF. What does countries SF mean?

R1C1 response

**We thank the reviewer for identifying this typographical error. We have corrected this sentence in the abstract so that it now reads (L32):**

“Substandard and falsified (SF) medicines are a neglected public health threat, with an estimated 7% of antimicrobials in low- and middle-income countries being SF.”

R1C2

Line 91 Graph: we reanalyzed data from seven clinical trials of ofloxacin as a treatment for typhoid: Is this clinical trial data from the same country as the population/API distribution included in the model? It's described as being from Vietnam, but one API distribution is from Bangladesh.

R1C2 response

**We agree that this needed to be clearer in the figure and surrounding text. The clinical trial data are from Vietnam, while the medicine quality survey data come from several countries. In the main analysis, the ofloxacin clinical dose-response relationship is estimated using the Vietnam trial data, and this dose-response relationship is then combined with the observed %API distributions from medicine quality surveys. These distributions are not intended to represent Vietnam specifically. Instead, they represent examples of observed fluoroquinolone quality distributions that can be combined with the estimated dose-response relationship to explore the potential effect of medicines with incorrect %API in settings with different levels of medicine quality.**

**We have amended the Methods to clarify that the clinical data and medicine quality data need not come from the same country, but that the interpretation of the population-level estimates depends on the representativeness of the medicine quality distribution used. We have added the following text (L178):**

“Note that the medicine quality surveys and clinical trials do not need to come from the same setting. The dose-response relationship is estimated from individual-level clinical data, while the %API distribution represents the medicine quality setting to which the framework is being applied. Population-level estimates should therefore be interpreted as applying to settings represented by the selected %API distribution, rather than necessarily to the country in which the clinical trials were conducted.”

R1C3

Line 129: probability to investigate....... There is no dot after probability.

R1C3 response

**We have added a full stop after “probability.”**

R1C4

How did you measured or calculated for treatment outcomes from Bangladesh in the model? It's known that its API is substandard (less than 60%). LINE 199, while the clinical trial used for modeling is from Vietnam.

R1C4 response

**We did not measure treatment outcomes in Bangladesh, and no Bangladeshi clinical outcome data were used in the model. Instead, the clinical dose-response relationship was estimated using the Vietnam ofloxacin trial data. The Bangladesh ciprofloxacin data were used only as a medicine quality distribution in a scenario analysis, because this was the distribution with the lowest observed %API values among the included random surveys.**

**We have clarified this in the Results. In particular, we now describe the Bangladesh analysis as a scenario analysis rather than as an estimate of treatment outcomes observed in Bangladesh (L254):**

“When we instead assumed that %API followed the Bangladesh ciprofloxacin distribution (lowest mean %API; Fig 3B), chosen to represent a low-%API scenario rather than a precise estimate of treatment outcomes in Bangladesh, we estimated a 52.6h (95% CrI: 8.84–116) increase in fever clearance time (Fig S2).”

R1C5

Line 246: When %API followed the ciprofloxacin distribution (mean <100% %; Fig 3B) Why does Fig 3B use SF Cipro survey distribution data as the API estimate when it actually uses clinical trial data from OFLO? -> This is discussed in the limitations section.

R1C5 response

**We agree with the reviewer that this point should be clearer before the limitations section. The reason for including the ciprofloxacin distribution is that there were very few random surveys of ofloxacin quality, while ciprofloxacin is also a fluoroquinolone and is currently recommended by the WHO AWaRe book for typhoid fever when the risk of fluoroquinolone resistance is low. We therefore used the ciprofloxacin %API distribution as a proxy and scenario analysis for a lower-mean fluoroquinolone %API distribution, not because we assume ciprofloxacin and ofloxacin are identical medicines.**

**We have revised the Results to make this interpretation explicit (L251):**

“Because only one eligible random survey reported ofloxacin %API, we also explored a scenario in which ofloxacin %API followed the distribution observed in ciprofloxacin surveys. This is chosen to represent a proxy fluoroquinolone medicine-quality scenario with lower average %API.”

R1C6

Line277- 278: insufficient doses does not mean that the medicines is substandard or falsified. How to explain the correlation with research titles related to the impact of substandard or counterfeit drugs?

R1C6 response

**We agree with the reviewer, an insufficient dose may occur for reasons unrelated to medicine quality, such as prescribing or adherence, and does not necessarily imply that a medicine is substandard or falsified. In our analysis, the clinical trial data are used to estimate how different effective ofloxacin doses affect outcomes. We then apply this estimated dose-response relationship to medicines with incorrect %API, because medicines containing less API than stated would deliver a lower effective dose if the same number of tablets were taken.**

**To avoid implying that every low dose is caused by SF medicines, we have revised the Discussion. The sentence now reads (L289):**

“We found that lower effective ofloxacin doses, such as those that would occur when medicines contain substantially less API than stated, can substantially worsen individual-level outcomes when infecting strains have high MIC (e.g., 1 mg/L), particularly for fever clearance times.”

R1C7

Line 297: The potential risks of SF fluoroquinolones are also likely to have increased over time as the fluoroquinolone MIC of S. Typhi increases. Why does the potential of fluoroquinolones to act as SF increase as the fluoroquinolone MIC of S. Typhi increases? Can you explain this further?

R1C7 response

**We did not intend to say that fluoroquinolones become more likely to be SF as MIC increases. Rather, we meant that our results suggest that the clinical consequences of receiving a fluoroquinolone with low %API are likely to be worse when the infecting strain has a higher MIC. We have revised this part of the Discussion to make the mechanism clearer (L310):**

“The potential clinical risks posed by SF fluoroquinolones are also likely to have increased as the fluoroquinolone MIC of S. Typhi has risen. For susceptible infections with low MICs, standard dosing may provide a sufficient margin that modest reductions in API have little effect on outcomes. For infections with high MICs, this margin is smaller, so medicines containing low %API are more likely to result in inadequate exposure and delayed clinical response.”

R1C8

Line 337 Our framework for quantifying the impact of SF antimicrobials demonstrates that the population-level impacts of SF antimicrobials on typhoid outcomes may be heightened when the MIC of S. Typhi is high and the SF antimicrobials have low %API.

So, the thought process is this: SF medicine for antibiotics is assessed based on the %API results. Disease severity is assessed by the MIC. The assumption is that when the %API is outside the normal range, it is insufficient to kill the bacteria, thus prolonging the patient's symptoms. For typhoid, there are two drugs: Cipro and Oflo. The SF survey results only show one study for Oflo, while there are seven studies for Cipro. Clinical trials were conducted only for Oflo, with seven studies.

Question: • Why is the SF Cipro survey discussed if it only uses clinical trial data for OFLO? -> This is discussed in the limitations section. • Is there any data on the relationship between %API and outcome, or the relationship between %API and the effect on MIC? If not, how is the impact of %API on MIC and clinical trial outcome calculated? Is it by estimating the %API to be a lower or higher dose? • The distribution of SF Meds Survey results is used to estimate the relative risk of outcome according to Step 3 in Figure 1. Estimate the population-level relative risk of outcome due to medicines with incorrect %API, but is the probability (in addition to the distribution range) of the SF drug %API based on that distribution considered in the estimate/weighting when calculating the relative risk?

R1C8 response

**We thank the reviewer for this helpful summary of the logic of the framework. We agree that several parts of this logic needed to be explained more clearly.**

**First, we discuss the ciprofloxacin surveys because only one eligible random survey reported ofloxacin %API, whereas more data were available for ciprofloxacin, and ciprofloxacin is also a fluoroquinolone used in typhoid treatment. The ciprofloxacin analyses are therefore intended as proxy/scenario analyses. We have clarified this earlier in the Results, as described in response to R1C5, and not only in the limitations.**

**Second, we do not have data that directly link %API to clinical outcomes at the individual patient level, nor do we model %API as changing the MIC of the infecting strain. MIC is treated as a pathogen susceptibility measure and an effect modifier of the dose-response relationship. The role of %API is to modify the effective dose received by the patient. For example, a medicine containing 80% API is assumed to deliver 80% of the labelled active ingredient, while a medicine containing 120% API is assumed to deliver 120% of the labelled active ingredient. We then use the clinical trial dose-response model to estimate the expected outcome at that lower or higher effective dose, for a given MIC.**

**We have added the following clarification to the Methods (L134):**

“Here we are treating MIC as an effect modifier of the dose-response relationship.”

**Third, the probability of different %API values is included in the Step 3 estimate. In the Methods equation, p(a) is the probability density of receiving each effective dose implied by the observed %API distribution. Thus, %API values that occur more often in the survey distribution contribute more to the population-level estimate than rare values. We have revised the text around the equation to make this weighting clearer (L178):**

“The integral averages over the full observed %API distribution. Values of %API that are more common in the medicine quality surveys have greater weight through p(a), while rare values contribute less to the population-level estimate.”

R1C9

Finding step 3: we estimated a 4.1h (90% CrI: 0.75–8.7) reduction in fever clearance time for non-susceptible infections with an MIC of 1 mg/L (line 243) -> This is only for the target dose of 52 mg/kg

R1C9 response

**We agree and have amended the Results to specify the target dose. The sentence now reads (L253):**

“When the target dose was 52 mg/kg and %API followed the ofloxacin distribution, we estimated a 4.1h (90% CrI: 0.75–8.7) reduction in fever clearance time for non-susceptible infections with an MIC of 1 mg/L compared to ofloxacin with 100% API.”

R1C10

Finding step 3: we estimated an 11h (90% CrI: 1.9–25) increase in fever clearance time for an MIC of 1 mg/L (line 246) -> This is only for the target dose of 52 mg/kg

R1C10 response

**We agree and have amended the Results to specify the target dose. The sentence now reads (L259):**

“When the target dose was 52 mg/kg and %API followed the ciprofloxacin distribution, we estimated an 11h (90% CrI: 1.9–25) increase in fever clearance time for infections with an MIC of 1 mg/L.”

Reviewer #2

R2C1

1. Analysis assumes that all antimicrobials administered in the clinical trials contained 100% API. Given that this assumption underpins the entire modeling framework, how do you justify its validity, and can you provide quantitative sensitivity analyses to demonstrate how violations of this assumption would affect your conclusions?

R2C1 response

**We agree that this is an important assumption and have expanded the Methods and Discussion to address it more directly. The original clinical trials did not report %API testing of the trial medicines, so we cannot verify that the administered tablets contained exactly 100% API. Our justification for the baseline assumption is that these were supervised clinical trials in which medicines were administered according to trial protocols, making large systematic deviations from labelled dose less likely than in routine care. However, we agree that this remains an assumption.**

**We have now added sensitivity analyses in which the effective doses in the clinical trial data were shifted to reflect possible systematic deviations from 100% API. Specifically, we repeated the analysis assuming that the clinical trial medicines contained 90% and 110% of the labelled API. These analyses shift the estimated dose-response relationship left or right, but the qualitative finding was unchanged: the estimated consequences of low %API were small for low-MIC infections and larger for high-MIC infections.**

**We have added the following text to the Methods (L127):**

“Because the %API of medicines used in the original trials was not reported, the baseline analysis assumed that trial medicines contained 100% of the stated API. To assess the sensitivity of our conclusions to this assumption, we repeated the main analysis after multiplying all trial doses by 0.9 and 1.1, corresponding to systematic trial-medicine API contents of 90% and 110% of the labelled amount.”

**We have also added the following text to the results section (L299):**

**“**Sensitivity analyses assuming that doses administered in the trial were either 90% or 110% of the stated API (Fig S12) or that doses were administered in whole tablet increments (Fig S13) gave similar results, suggesting that the main qualitative conclusions are not sensitive to these assumptions. As some of the variability in dosing is lost when assuming whole tablet increments, there is increased uncertainty in these results (Fig S13).**”**

**We have also expanded the limitations section (L342):**

“We examined this in a sensitivity analysis, assuming medicines were allocated randomly and the distribution of %API did not change substantially between studies, and found that assuming that all medicines had 100% API is unlikely to have qualitatively changed our findings.”

R2C2

2. The clinical data are restricted to Vietnam (1992–2001). How can the findings be considered applicable to current global health contexts, particularly in sub-Saharan Africa, where antimicrobial resistance patterns and treatment practices differ substantially?

R2C2 response

**We agree with the reviewer that the clinical data are not globally representative of current typhoid treatment practice. We have revised the manuscript to avoid implying that the Vietnam trials directly represent current global typhoid treatment contexts. Instead, we present this as an application of a general framework using the best available individual-level data linking ofloxacin dose, MIC, and clinical outcomes.**

**The framework separates two components: the dose-response relationship estimated from clinical data, and the medicine quality distribution used for population-level inference. The Vietnam trial data are used to estimate the former. The latter can be updated for different regions as local medicine quality data become available. We also stratify the estimates by MIC because one of the main reasons that findings may differ across settings is variation in fluoroquinolone susceptibility. This is particularly relevant to modern contexts where MIC distributions differ substantially from those in the original trials.**

**We have added the following text to the Discussion:**

“The clinical trials used here were conducted in Vietnam between 1992 and 2001, and the estimated dose-response relationship may not capture all features of current typhoid treatment in other regions, including sub-Saharan Africa. Treatment practices, patient populations, co-morbidities, health-care access, and antimicrobial resistance patterns may differ. We therefore interpret this analysis as an application of a general framework rather than as a direct estimate for every contemporary setting. The framework can be updated when individual-level dose-response data from other regions or more recent time periods become available. Until such data are available, the most relevant way to explore differences between resistance settings is through the MIC-stratified analyses, which show that the effect of low %API is most pronounced when the infecting strain has reduced fluoroquinolone susceptibility.”

R2C3

3. The medicine quality dataset appears sparse, heterogeneous, and geographically uneven. How do you address potential selection bias and lack of representativeness, and what is the impact of these limitations on the robustness of your estimates?

R2C3 response

**We agree that the medicine quality data available for this application are sparse and geographically uneven, particularly for ofloxacin. However, our objective was not to estimate a globally representative distribution of fluoroquinolone quality. Rather, the framework is designed to combine a clinical dose-response relationship with any observed distribution of medicine quality. The included surveys should therefore be interpreted as examples of observed %API distributions that can be incorporated into the framework, rather than as definitive estimates of global fluoroquinolone quality. We have clarified this interpretation in the Discussion (see R2C2).**

R2C4

4. Given that ciprofloxacin data are used as a proxy for ofloxacin due to limited availability, how do you justify this substitution pharmacologically and epidemiologically, and have you assessed the uncertainty introduced by this assumption?

R2C4 response

**We agree that the use of ciprofloxacin quality data requires careful interpretation. Because only one eligible random survey reported ofloxacin %API, we additionally explored ciprofloxacin quality distributions as alternative fluoroquinolone medicine-quality scenarios. These analyses should not be interpreted as evidence that ciprofloxacin and ofloxacin have identical quality distributions. Importantly, the clinical dose-response model remains based entirely on ofloxacin trial data; the ciprofloxacin surveys are used only to represent alternative observed fluoroquinolone quality distributions within Step 1 of the framework. We have clarified this point in the Discussion (see R2C2).**

R2C5

5. The manuscript lacks sufficient detail on model diagnostics (e.g., convergence metrics, posterior predictive checks). Can you provide comprehensive diagnostic results to demonstrate that the Bayesian models are well-calibrated and robust?

R2C5 response

**We agree that more diagnostic information is needed. We have added a supplementary section reporting convergence diagnostics and posterior predictive checks for all primary and sensitivity models. Specifically, we now report R-hat values, effective sample sizes, visual inspection of trace plots, divergent transitions, and posterior predictive checks for fever clearance time and treatment failure. All R-hat values were <1.01, bulk and tail effective sample sizes were >2000, and no divergent transitions were observed.**

**We have added the following text to the Methods:**

“We assessed convergence using R-hat, bulk and tail effective sample sizes, trace plots, and the number of divergent transitions. We assessed model calibration using posterior predictive checks comparing replicated data generated from the posterior predictive distribution with the observed distributions of fever clearance time and treatment failure.”

**We have added the following text to the Results:**

“All primary models showed satisfactory convergence diagnostics, with R-hat values <1.01, effective sample sizes above 2000, and no divergent transitions (Table S4). Posterior predictive checks indicated that the models captured the main features of the observed fever clearance time and treatment failure data. Diagnostic plots are shown in Figs S13-16.”

R2C6

6. Approximately 5.9% of observations were excluded due to missing MIC values. How might this exclusion bias your findings, and have you explored imputation methods or sensitivity analyses to assess the impact?

R2C6 response

**We agree that excluding patients with missing MIC values could introduce bias. Approximately 5.9% of observations were excluded for this reason. Given the relatively small proportion of excluded observations, we do not expect this to be a major source of bias. We considered imputation approaches but did not pursue them because MIC was a pre-specified effect modifier of the dose-response relationship and could not be derived reliably from other recorded variables.**

**We have added the following text to the limitations section (L375):**

“Approximately 5.9% of observations were excluded because MIC measurements were unavailable, which may introduce some degree of selection bias. However, given the relatively small proportion of excluded observations, any resulting bias is unlikely to alter the overall conclusions. Because MIC was a key effect modifier and directly measured microbiological characteristic, imputation would have required strong assumptions and was therefore not performed.”

R2C7

7. The reconstruction of the administered dose based on tablet rounding assumptions introduces potential measurement error. How have you quantified and accounted for this uncertainty in your models?

R2C7 response

**We agree that the exact administered dose is not directly observed for all trials. However, our reconstruction was intended to reduce, rather than introduce, measurement error relative to assuming that all participants received exactly their target mg/kg dose. Because dosing was constrained by tablet formulation, the target dose could not generally have been administered exactly. The primary analysis therefore used the most plausible reconstruction, based on 200 mg tablets administered in half-tablet increments. To quantify sensitivity to this assumption, we repeated the fever-clearance-time analysis using a more conservative alternative in which doses were reconstructed assuming whole-tablet increments. The qualitative conclusions were unchanged.**

**We have added the following text to the Methods (L126):**

“ We assessed sensitivity to the tablet-rounding assumption by repeating the analysis assuming that doses were administered in whole-tablet increments.”

**We have also added the following text to the results section (L299):**

“Sensitivity analyses assuming that doses administered in the trial were either 90% or 110% of the stated API (Fig S12) or that doses were administered in whole tablet increments (Fig S13) gave similar results, suggesting that the main qualitative conclusions are not sensitive to these assumptions. As some of the variability in dosing is lost when assuming whole tablet increments, there is increased uncertainty in these results (Fig S13).”

R2C8

8. Some results rely on extrapolation beyond observed dose ranges. How do you ensure that these extrapolations are methodologically valid and not driving key conclusions, particularly in low-dose scenarios?

R2C8 response

**We agree that extrapolation beyond the observed dose range is an important limitation. We have revised the manuscript to distinguish clearly between results supported by interpolation within the observed trial dose range and results requiring extrapolation.**

**We have also expanded the Discussion (L390):**

“Results involving very low %API values should be interpreted cautiously because of this extrapolation.”

R2C9

9. The underlying individual-level clinical data are not fully publicly available, which may not comply with PLOS data-sharing requirements. How do you plan to ensure full reproducibility of results, and can the data be deposited in a controlled-access repository?

R2C9 response

**We agree that reproducibility and data availability are important. The individual-level clinical trial data are anonymised historical trial data, and we are not able to make them fully public. However, we have made the analysis code available and have generated synthetic data with the same statistical properties so that the full workflow can be run and checked.**

R2C10

10. The reported effects on fever clearance time are relatively modest in absolute terms. Could you better justify the clinical and public health significance of these findings and clarify how they meaningfully inform policy or intervention strategies?

R2C10 response

**We have added the following text to the Discussion:**

“Although some estimated mean changes in fever clearance time were modest, these effects may still be clinically meaningful because fever clearance time reflects the duration of symptomatic illness. Moreover, larger effects were observed among patients infected with strains with high MICs and in low-%API scenarios. The framework is therefore useful for identifying settings in which poor medicine quality is most likely to translate into worse clinical outcomes.”
