# Supplementary material for: A novel framework for quantifying the clinical impact of substandard and falsified antimicrobials: Application to typhoid fever
Source: PLOS Glob Public Health. 2026 Aug 31;6(8):e0007207. doi: 10.1371/journal.pgph.0007207 (PMC13529222; doi:10.1371/journal.pgph.0007207)
Supplement: Peer review history

# Peer review: Decision Letter 0

**DOI:** 10.1371/journal.pgph.0007207.r001

**Authors:** Kiatichai Faksri

1 Apr 2026

PGPH-D-25-03990

A novel framework for quantifying the clinical impact of substandard and falsified antimicrobials: application to typhoid fever.

PLOS Global Public Health

Dear Dr. Cavany,

Thank you for submitting your manuscript to PLOS Global Public Health. After careful consideration, we feel that it has merit but does not fully meet PLOS Global Public Health’s publication criteria as it currently stands. Therefore, we invite you to submit a revised version of the manuscript that addresses the points raised during the review process.

Please submit your revised manuscript by May 01 2026 11:59PM. If you will need more time than this to complete your revisions, please reply to this message or contact the journal office at globalpubhealth@plos.org. When you're ready to submit your revision, log on to https://www.editorialmanager.com/pgph/ and select the 'Submissions Needing Revision' folder to locate your manuscript file.

Please include the following items when submitting your revised manuscript:

- A letter that responds to each point raised by the editor and reviewer(s). You should upload this letter as a separate file labeled 'Response to Reviewers'.
- A marked-up copy of your manuscript that highlights changes made to the original version. You should upload this as a separate file labeled 'Revised Manuscript with Track Changes'.
- An unmarked version of your revised paper without tracked changes. You should upload this as a separate file labeled 'Manuscript'.

Guidelines for resubmitting your figure files are available below the reviewer comments at the end of this letter.

As the corresponding author, your ORCID iD is verified in the submission system and will appear in the published article. PLOS supports the use of ORCID, and we encourage all coauthors to register for an ORCID iD and use it as well. Please encourage your coauthors to verify their ORCID iD within the submission system before final acceptance, as unverified ORCID iDs will not appear in the published article. *Only*  the individual author can complete the verification step; PLOS staff *cannot*  verify ORCID iDs on behalf of authors.

We look forward to receiving your revised manuscript.

Kind regards,

Kiatichai Faksri, Ph.D

Academic Editor

PLOS Global Public Health

**Journal Requirements:**

1. Please amend your detailed Financial Disclosure statement. This is published with the article. It must therefore be completed in full sentences and contain the exact wording you wish to be published.

i. Please clarify all sources of financial support for your study. List the grants, grant numbers, and organizations that funded your study, including funding received from your institution. Please note that suppliers of material support, including research materials, should be recognized in the Acknowledgements section rather than in the Financial Disclosure.

ii. State the initials, alongside each funding source, of each author to receive each grant. For example: "This work was supported by the National Institutes of Health (####### to AM; ###### to CJ) and the National Science Foundation (###### to AM)."

iii. State what role the funders took in the study. If the funders had no role in your study, please state: “The funders had no role in study design, data collection and analysis, decision to publish, or preparation of the manuscript.”

iv. If any authors received a salary from any of your funders, please state which authors and which funders.

2. We ask that a manuscript source file is provided at Revision. Please upload your manuscript file as a .doc, .docx, .rtf or .tex.

3. Please ensure that your Ethics Statement is available in its entirety at the beginning of your Methods section, under a subheading 'Ethics Statement'.

4. Please upload separate figure files in .tif or .eps format. Also, remove the figures from your manuscript file but keep the legends.

For more information about figure files please see our guidelines:

https://journals.plos.org/globalpublichealth/s/figures

https://journals.plos.org/globalpublichealth/s/figures#loc-file-requirements

5. We have noticed that you have uploaded Supporting Information files, but you have not included a list of legends. Please add a full list of legends for your Supporting Information files after the references list.

6. Please note that your Data Availability Statement is currently missing the DOI/accession number of each dataset OR a direct link to access each database. If your manuscript is accepted for publication, you will be asked to provide these details on a very short timeline. We therefore suggest that you provide this information now, though we will not hold up the peer review process if you are unable.

If the reviewer comments include a recommendation to cite specific previously published works, please review and evaluate these publications to determine whether they are relevant and should be cited. There is no requirement to cite these works unless the editor has indicated otherwise.

Please review your reference list to ensure that it is complete and correct. If you have cited papers that have been retracted, please include the rationale for doing so in the manuscript text, or remove these references and replace them with relevant current references. Any changes to the reference list should be mentioned in the rebuttal letter that accompanies your revised manuscript. If you need to cite a retracted article, indicate the article’s retracted status in the References list and also include a citation and full reference for the retraction notice.

**Additional Editor Comments (if provided):**

Dear Dr. Cavany,

Thank you for submitting your manuscript, “A novel framework for quantifying the clinical impact of substandard and falsified antimicrobials: application to typhoid fever.”

Following peer review, your manuscript is suitable for publication pending minor revisions. Please address the reviewers’ comments to improve clarity and rigor.

Kindly submit:

A revised manuscript with changes clearly indicated

A point-by-point response to reviewer comments

We look forward to receiving your revision.

Sincerely,

Prof. Kiatichai Faksri

Academic Editor,

PLOS Global Public Health

[Note: HTML markup is below. Please do not edit.]

Reviewers' comments:

Reviewer's Responses to Questions

**Comments to the Author**

1. Does this manuscript meet PLOS Global Public Health’s publication criteria?

Reviewer #1: Yes

Reviewer #2: Yes

**********

2. Has the statistical analysis been performed appropriately and rigorously?

Reviewer #1: Yes

Reviewer #2: Yes

**********

3. Have the authors made all data underlying the findings in their manuscript fully available (please refer to the Data Availability Statement at the start of the manuscript PDF file)?

The PLOS Data policy

Reviewer #1: Yes

Reviewer #2: Yes

**********

4. Is the manuscript presented in an intelligible fashion and written in standard English?

Reviewer #1: Yes

Reviewer #2: Yes

**********

**Reviewer #1:** This study is very interesting, and there are still few publications that link medicines quality with treatment outcomes. However, there are some comments to improve the information and discussion in the paper.

The comments are as follows:

* Line 33: of antimicrobials in low- and middle-income countries SF.

What does countries SF mean?

* Line 91 Graph: we reanalyzed data from seven clinical trials of ofloxacin as a treatment for typhoid: Is this clinical trial data from the same country as the population/API distribution included in the model? It's described as being from Vietnam, but one API distribution is from Bangladesh.

* Line 129: probability to investigate.......

There is no dot after probability.

* How did you measured or calculated for treatment outcomes from Bangladesh in the model? It's known that its API is substandard (less than 60%). LINE 199, while the clinical trial used for modeling is from Vietnam.

* Line 246: When %API followed the ciprofloxacin distribution (mean <100% %; Fig 3B)

Why does Fig 3B use SF Cipro survey distribution data as the API estimate when it actually uses clinical trial data from OFLO? -> This is discussed in the limitations section.

* Line277- 278: insufficient doses does not mean that the medicines is substandard or falsified. How to explain the correlation with research titles related to the impact of substandard or counterfeit drugs?

* Line 297: The potential risks of SF fluoroquinolones are also likely to have increased over time as the fluoroquinolone MIC of S. Typhi increases.

Why does the potential of fluoroquinolones to act as SF increase as the fluoroquinolone MIC of S. Typhi increases? Can you explain this further?

* Line 337 Our framework for quantifying the impact of SF antimicrobials demonstrates that the

population-level impacts of SF antimicrobials on typhoid outcomes may be heightened when the MIC of S. Typhi is high and the SF antimicrobials have low %API.

So, the thought process is this: SF medicine for antibiotics is assessed based on the %API results. Disease severity is assessed by the MIC. The assumption is that when the %API is outside the normal range, it is insufficient to kill the bacteria, thus prolonging the patient's symptoms.

For typhoid, there are two drugs: Cipro and Oflo. The SF survey results only show one study for Oflo, while there are seven studies for Cipro. Clinical trials were conducted only for Oflo, with seven studies.

Question:

• Why is the SF Cipro survey discussed if it only uses clinical trial data for OFLO? -> This is discussed in the limitations section.

• Is there any data on the relationship between %API and outcome, or the relationship between %API and the effect on MIC? If not, how is the impact of %API on MIC and clinical trial outcome calculated? Is it by estimating the %API to be a lower or higher dose?

• The distribution of SF Meds Survey results is used to estimate the relative risk of outcome according to Step 3 in Figure 1. Estimate the population-level relative risk of outcome due to medicines with incorrect %API, but is the probability (in addition to the distribution range) of the SF drug %API based on that distribution considered in the estimate/weighting when calculating the relative risk?

* Finding step 3: we estimated a 4.1h (90% CrI: 0.75–8.7) reduction in fever clearance time for non-susceptible infections with an MIC of 1 mg/L (line 243) -> This is only for the target dose of 52 mg/kg

* Finding step 3: we estimated an 11h (90% CrI: 1.9–25) increase in fever clearance time for an MIC of 1 mg/L (line 246) -> This is only for the target dose of 52 mg/kg

**Reviewer #2:**  1. Analysis assumes that all antimicrobials administered in the clinical trials contained 100% API. Given that this assumption underpins the entire modeling framework, how do you justify its validity, and can you provide quantitative sensitivity analyses to demonstrate how violations of this assumption would affect your conclusions?

2. The clinical data are restricted to Vietnam (1992–2001). How can the findings be considered applicable to current global health contexts, particularly in sub-Saharan Africa, where antimicrobial resistance patterns and treatment practices differ substantially?

3. The medicine quality dataset appears sparse, heterogeneous, and geographically uneven. How do you address potential selection bias and lack of representativeness, and what is the impact of these limitations on the robustness of your estimates?

4. Given that ciprofloxacin data are used as a proxy for ofloxacin due to limited availability, how do you justify this substitution pharmacologically and epidemiologically, and have you assessed the uncertainty introduced by this assumption?

5. The manuscript lacks sufficient detail on model diagnostics (e.g., convergence metrics, posterior predictive checks). Can you provide comprehensive diagnostic results to demonstrate that the Bayesian models are well-calibrated and robust?

6. Approximately 5.9% of observations were excluded due to missing MIC values. How might this exclusion bias your findings, and have you explored imputation methods or sensitivity analyses to assess the impact?

7. The reconstruction of the administered dose based on tablet rounding assumptions introduces potential measurement error. How have you quantified and accounted for this uncertainty in your models?

8. Some results rely on extrapolation beyond observed dose ranges. How do you ensure that these extrapolations are methodologically valid and not driving key conclusions, particularly in low-dose scenarios?

9. The underlying individual-level clinical data are not fully publicly available, which may not comply with PLOS data-sharing requirements. How do you plan to ensure full reproducibility of results, and can the data be deposited in a controlled-access repository?

10. The reported effects on fever clearance time are relatively modest in absolute terms. Could you better justify the clinical and public health significance of these findings and clarify how they meaningfully inform policy or intervention strategies?

**********

what does this mean?). If published, this will include your full peer review and any attached files.

**Do you want your identity to be public for this peer review?**  If you choose “no”, your identity will remain anonymous but your review may still be made public.

For information about this choice, including consent withdrawal, please see our Privacy Policy

Reviewer #1: No

Reviewer #2: **Yes:** NGALE EBONG Darlintine Ejede

**********

[NOTE: If reviewer comments were submitted as an attachment file, they will be attached to this email and accessible via the submission site. Please log into your account, locate the manuscript record, and check for the action link "View Attachments". If this link does not appear, there are no attachment files.]

**Figure Resubmissions:**

# Author comment: Author response to Decision Letter 1

**DOI:** 10.1371/journal.pgph.0007207.r002

23 Jul 2026

Attachment Submitted filename: typhoid_direct_impacts_response_to_reviewers_20260507.docx

# Peer review: Decision Letter 1

**DOI:** 10.1371/journal.pgph.0007207.r003

**Authors:** Kiatichai Faksri

11 Aug 2026

A novel framework for quantifying the clinical impact of substandard and falsified antimicrobials: application to typhoid fever.

PGPH-D-25-03990R1

Dear Dr. Cavany,

We are pleased to inform you that your manuscript 'A novel framework for quantifying the clinical impact of substandard and falsified antimicrobials: application to typhoid fever.' has been provisionally accepted for publication in PLOS Global Public Health.

Before your manuscript can be formally accepted you will need to complete some formatting changes, which you will receive in a follow up email. A member of our team will be in touch with a set of requests.

Please note that your manuscript will not be scheduled for publication until you have made the required changes, so a swift response is appreciated.

IMPORTANT: The editorial review process is now complete. PLOS will only permit corrections to spelling, formatting or significant scientific errors from this point onwards. Requests for major changes, or any which affect the scientific understanding of your work, will cause delays to the publication date of your manuscript.

If your institution or institutions have a press office, please notify them about your upcoming paper to help maximize its impact. If they'll be preparing press materials, please inform our press team as soon as possible -- no later than 48 hours after receiving the formal acceptance. Your manuscript will remain under strict press embargo until 2 pm Eastern Time on the date of publication. For more information, please contact globalpubhealth@plos.org.

Thank you again for supporting Open Access publishing; we are looking forward to publishing your work in PLOS Global Public Health.

Best regards,

Kiatichai Faksri, Ph.D

Academic Editor

PLOS Global Public Health

***********************************************************

Reviewer Comments (if any, and for reference):

Reviewer's Responses to Questions

**Comments to the Author**

Reviewer #1: All comments have been addressed

Reviewer #2: All comments have been addressed

**********

publication criteria?

Reviewer #1: Yes

Reviewer #2: Yes

**********

3. Has the statistical analysis been performed appropriately and rigorously?

Reviewer #1: Yes

Reviewer #2: Yes

**********

4. Have the authors made all data underlying the findings in their manuscript fully available (please refer to the Data Availability Statement at the start of the manuscript PDF file)?

The PLOS Data policy

Reviewer #1: Yes

Reviewer #2: Yes

**********

5. Is the manuscript presented in an intelligible fashion and written in standard English?

Reviewer #1: Yes

Reviewer #2: Yes

**********

Reviewer #1: All the comments and suggestion from the previous manuscript have been addressed and revised accordingly.

Reviewer #2: (No Response)

**********

what does this mean?). If published, this will include your full peer review and any attached files.

**Do you want your identity to be public for this peer review?**  If you choose “no”, your identity will remain anonymous but your review may still be made public.

For information about this choice, including consent withdrawal, please see our Privacy Policy

Reviewer #1: No

Reviewer #2: **Yes:** NGALE EBONG Darlintine Ejede

**********
